# Supplementary material for: Early Postnatal Exposure to Midazolam Causes Lasting Histological and Neurobehavioral Deficits via Activation of the mTOR Pathway
Source: Int J Mol Sci. 2024 Jun 19;25(12):6743. doi: 10.3390/ijms25126743 (PMC11203812; doi:10.3390/ijms25126743)
Supplement: Supplementary file 1 [file ijms-25-06743-s001.zip › ijms-3018241-supplementary.pdf]

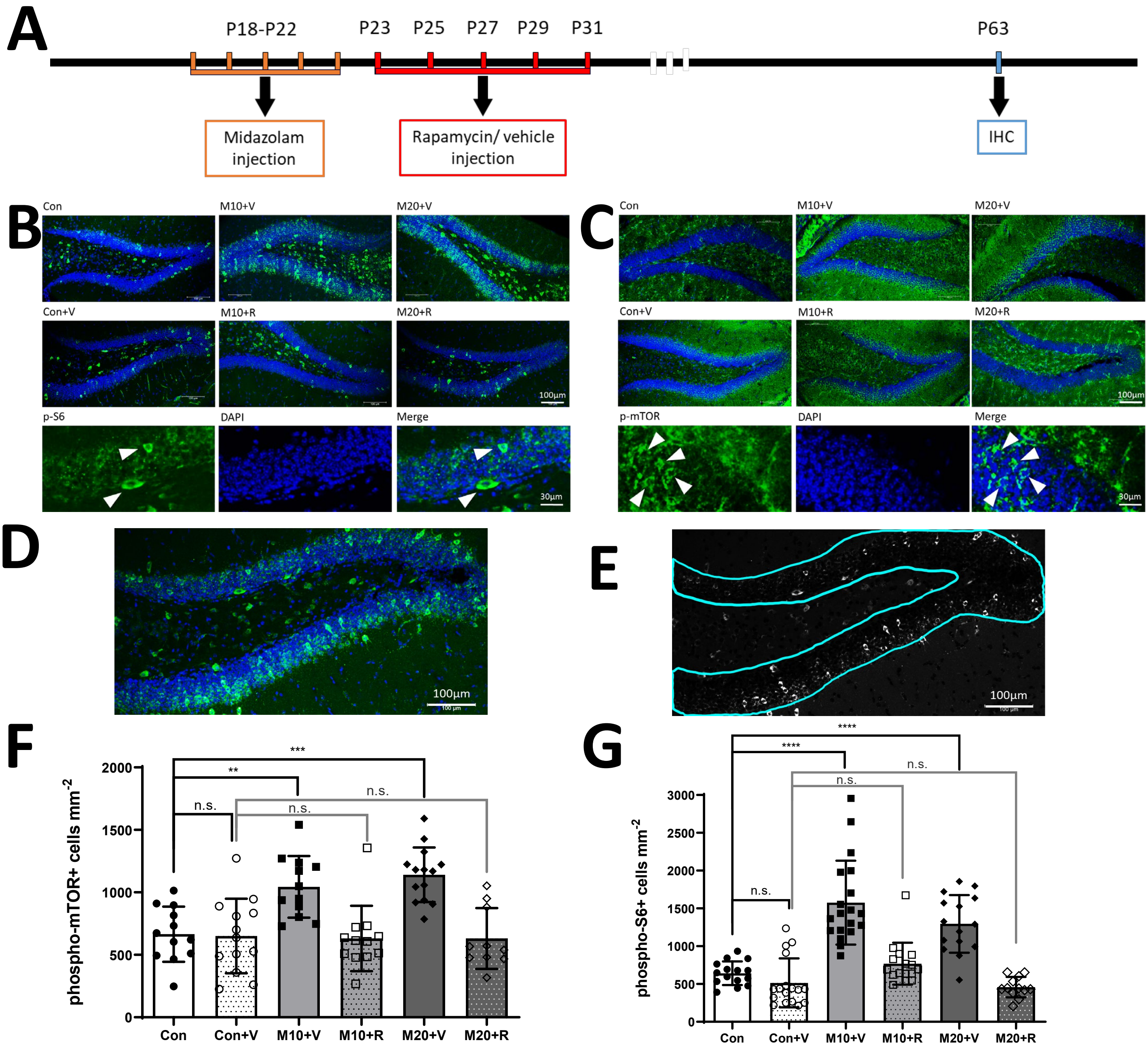

Supplement Figure S1. Midazolam exposure leads to aberrant activation of the mTOR signaling pathway in the mouse dentate gyrus.

(A). Schematic representation of the experimental timeline for immunohistochemistry.

(B-C). Representative confocal images of phospho-S6 and phospho-mTOR expression in exposed DG neurons at P63 (white arrows point at the qualified positive cells).

(D-E). Representative confocal images for image analysis.

(F-G). Downstream markers of mTOR pathway, phospho-mTOR and phospho-S6 expression increased after midazolam exposure and using the inhibitor of mTOR pathway can prevent this increase. (n=12-19 per group. **p<0.01, ***p<0.001, ****p<0.0001 ANOVA, n.s. indicates no significant difference compared to the control group)

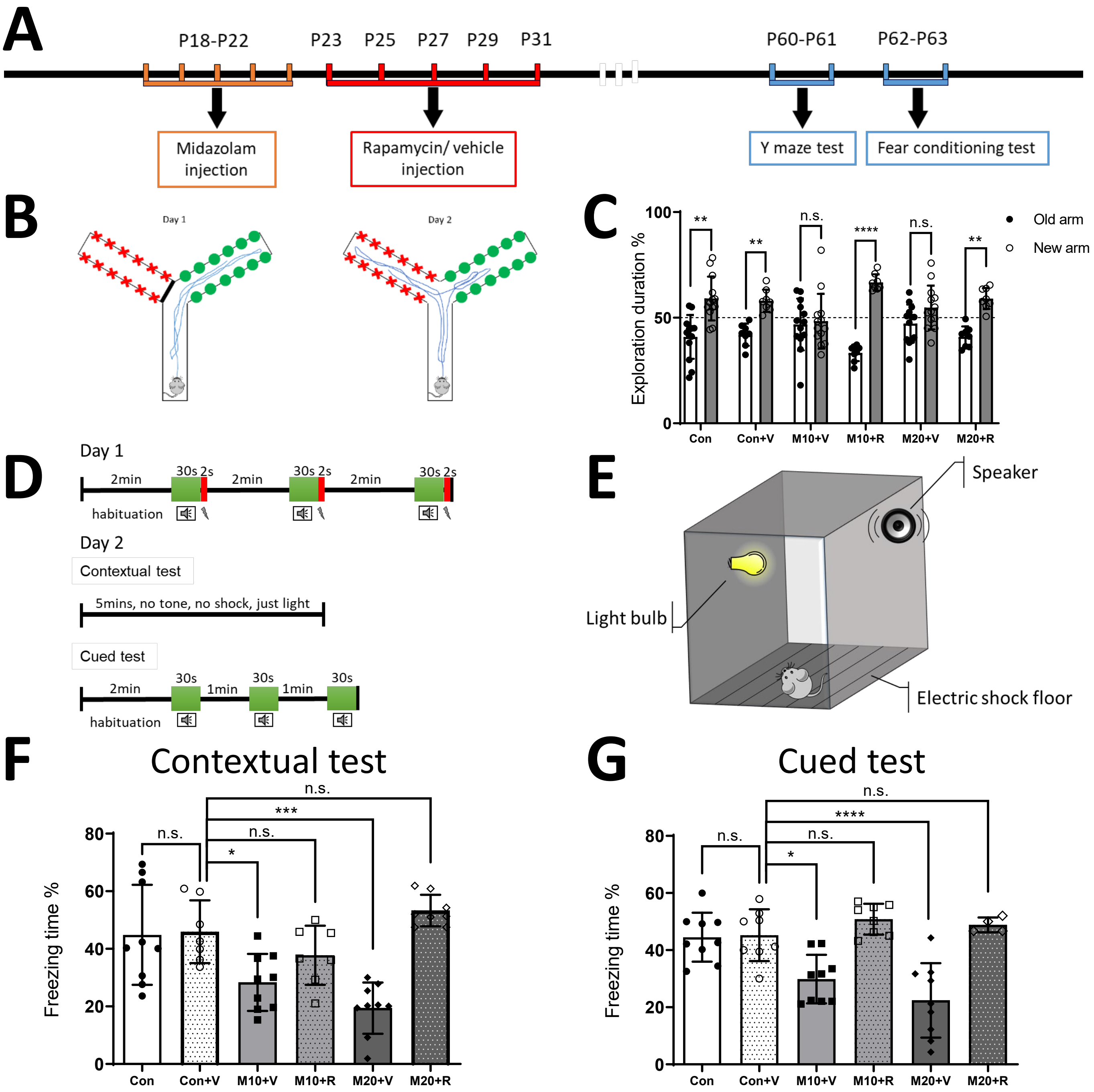

Supplement Figure S2. Inhibition of mTOR activities rescues deficits in the dentate gyrus dependent spatial learning and memory after midazolam exposure.

(A). Schematic representation of the experimental timeline for behavior tests.

(B-C). In the Y-maze test (diagram in **B**), control animals exhibit normal performance, spending significantly longer times in the novel arm. Midazolam sedated animals (both low and high dose groups) did not differ significantly in time spent in the novel and old arms, suggesting impaired learning and memory. Given rapamycin treatment turned the results back to normal (**B**). (n=8-13 per group, $**p<0.01$, $****p<0.0001$ *t*-test, n.s. indicates no significant difference compared to the control group)

(D-G). In the fear conditioning test (diagram in **D,E**), as compared to controls, the midazolam sedated mice (both low and high dose groups) showed significantly reduced freezing time percentages in the contextual test compared (**F**) and the cued test (**G**). After rapamycin treatment, the midazolam exposed animals showed no significant different compared to the control group (n=7-9 per group. $*p<0.05$, $**p<0.01$, $***p<0.001$, $****p<0.0001$ ANOVA, n.s. indicates no significant difference)
